# An evaluation of clinical treatment of convergence insufficiency for children with reading difficulties

**DOI:** 10.1186/1471-2415-11-21

**Published:** 2011-08-11

**Authors:** Wolfgang A Dusek, Barbara K Pierscionek, Julie F McClelland

**Affiliations:** 1 Vision Science Research Group, School of Biomedical Sciences, University of Ulster, Coleraine, Co. Londonderry, UK, BT52 1SA

## Abstract

**Background:**

The present study investigates two different treatment options for convergence insufficiency CI for a group of children with reading difficulties referred by educational institutes to a specialist eye clinic in Vienna.

**Methods:**

One hundred and thirty four subjects (aged 7-14 years) with reading difficulties were referred from an educational institute in Vienna, Austria for visual assessment. Each child was given either 8Δ base-in reading spectacles (n = 51) or computerised home vision therapy (HTS) (n = 51). Thirty two participants refused all treatment offered (clinical control group). A full visual assessment including reading speed and accuracy were conducted pre- and post-treatment.

**Results:**

Factorial analyses demonstrated statistically significant changes between results obtained for visits 1 and 2 for total reading time, reading error score, amplitude of accommodation and binocular accommodative facility (within subjects effects) (p < 0.05). Significant differences were also demonstrated between treatment groups for total reading time, reading error score and binocular accommodative facility (between subjects effects) (p < 0.05).

**Conclusions:**

Reading difficulties with no apparent intellectual or psychological foundation may be due to a binocular vision anomaly such as convergence insufficiency. Both the HTS and prismatic correction are highly effective treatment options for convergence insufficiency. Prismatic correction can be considered an effective alternative to HTS.

## Background

It is well documented that children with reading difficulties are at a greater risk for anomalies of visual function and asthenopic symptoms than their peers without reading difficulties [1-3]. Reading difficulties are significantly related to various aspects of visual function including refractive error and binocular vision status [4-9]. Dusek et al [10] demonstrated differences in visual status between a large group of children with reading difficulties (n = 825) and a clinical control group (n = 328) in terms of visual acuity, ocular posture, accommodation, reading speed and convergence. One of the most significant findings was the high proportion of children with reading difficulties who demonstrated convergence insufficiency (CI) (18.2%) [10]. This is a common binocular vision disorder that frequently underpins a wide range of asthenopic symptoms in both adults and children [11-13].

Although a substantial volume of literature that describes various different treatments options for CI exists, progress is hampered by inconsistencies regarding the most appropriate treatment and a paucity of suitable treatment options for children with reading difficulties [14-17]. Evaluation of the use of base-in prism spectacles for CI is also limited [18,19]. To the best of the authors' knowledge, no controlled studies linking CI and reading difficulties have been undertaken on large European populations and certainly no previous studies have been conducted on Austrian school children.

The Austrian approach to detecting, evaluating and treating reading, writing and other learning difficulties is holistic. Children in Austria undergo regular health examinations and those who have difficulties with reading and writing are referred routinely by their teacher or parent to educational institutions for an assessment of their academic status. A range of standardised tests are conducted by educational psychologists to determine the cause(s) that may impede learning. The tests include intelligence assessments to investigate whether the difficulties arise from a reduced level of intelligence (low IQ) or whether there are any other underlying factors. In Vienna, these assessments are carried out in one of three educational institutes (Holistic Institut, Förderpädagogisches Zentrum and Gesundheits Zentrum). A significant proportion of these children are found to have a normal or above normal level of intellectual ability despite their reading and writing difficulties. These particular children are all referred to a single specialist clinician (WD) for a full assessment of visual status. Given that over a quarter of the Austrian population resides in Vienna and that this specialist clinic also accepts referrals from other regions of Austria, the database of children examined in this clinic is representative of the country as a whole and is characteristic of a European population of school children.

The present study is the first controlled study on a large homogenous European population examined by a single specialist. It investigates two different treatment options for CI for a group of children with reading difficulties referred by the aforementioned educational institutes to the specialist eye clinic in Vienna.

## Methods

### Subjects

Initially 1001 subjects were referred from three educational institutes in Vienna, Austria diagnosed with difficulties in reading and writing that could not be attributed to a learning difficulty. All subjects had been assessed by an educational psychologist and had an IQ (intelligence quotient) over 70. Subjects with ocular pathology (e.g. cataract, glaucoma, strabismus) were excluded from the study (n = 11) and referred for ophthalmological investigation. The visual status of a subset of this population has previously been described in detail [10]. One hundred and thirty four subjects were diagnosed with CI.

CI was confirmed if a subject demonstrated all of the first three clinical signs below. CI was also identified if subjects demonstrated at least two of the first three clinical signs with at least one other additional sign (point 4 and/or 5);

1. A near point of convergence (NPC) greater than 6 cm

2. Exophoria at both near and distance which was at least 6 prism dioptres more at near than at distance.

3. A low accommodative convergence to accommodation ratio (AC/A) (< 2:1)

4. A binocular accommodative facility of less than 6 cycles per minute using (+2.00/-2.00 flipper lenses) and a monocular accommodative facility better than 10 cycles per minute

5. Reduced vergence facility less than 6 cycles per minute (using base-out prism)

**Figure 1:**
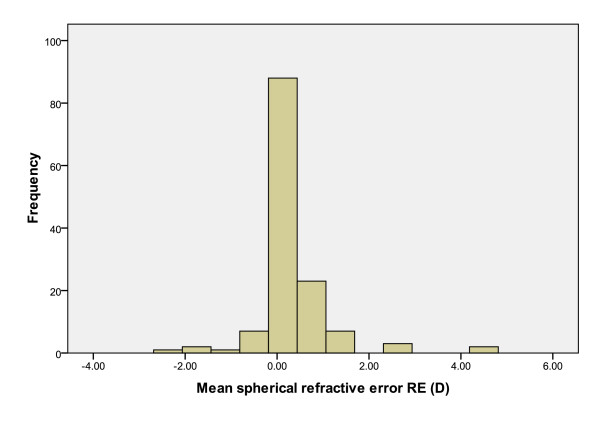
**Mean spherical equivalent refractive error of subjects with convergence insufficiency**.

All subjects with CI and reading difficulties (n = 134) attended for a second assessment four weeks after the first visit. Table 1 details the age and gender distribution of the subjects and Figure 1 illustrates the refractive error profile of the group.

**Table 1:** Age and gender distribution of the subject group

Age (years)	Number (n) (%)		Percentage (%)
	**Female**	**Male**	

**7**	5 (3.73)	10 (7.46)	11.2

**8**	17 (12.69)	18 (13.43)	26.1

**9**	14 (10.45)	14 (10.45)	20.9

**10**	6 (4.48)	12 (8.96)	13.4

**11**	8 (5.97)	13 (9.70)	15.7

**12**	2 (1.49)	7 (5.22)	6.7

**13**	2 (1.49)	3 (2.24)	3.7

**14**	0 (0)	3 (2.24)	2.2

All subjects in the present study were attending mainstream schools. Ethical approval for the study was obtained from the University of Ulster Research Ethics Committee and the study adhered to the Tenets of the Declaration of Helsinki. Written informed consent was obtained from all parents of the subjects included in the study.

### Procedure

#### Intervention

Two different types of treatment for CI were employed: a computerised home visual therapy system (HTS) and reading glasses without additional refractive power but with 8Δ base-in.

Details of the two treatment options were explained to all subjects and their parents and subjects were free to choose either treatment option. This study was not a randomised controlled trial, however, inclusion of a subject in either treatment group had no dependency on degree of CI, refractive error, age, initial measures of reading speed, reading accuracy, binocular accommodative facility, amplitude of accommodation, near point of convergence, ocular posture, MEM retinoscopy or vergence facility. This was confirmed by statistical analysis (one way ANOVA (p > 0.05). Thirty-two subjects refused both types of treatment offered and agreed to return for a subsequent assessment four weeks later as a control group for the study. Sample sizes were based on available clinical data. The 8Δ base-in spectacle group included 51 subjects and the HTS groups also included 51 subjects.

The majority of subjects in the 8Δ base-in group did not have a significant distance refractive error and were issued with spectacles for near vision only. Five subjects (two myopic and three hyperopic), were issued with Franklin split bifocals with the 8Δ base-in incorporated into the near portion of the spectacles.

Subjects issued with the reading glasses (8Δ base-in) were advised to use the spectacles for all near vision tasks that were greater than five minutes duration [18]. Subjects given the HTS were given written and verbal information on installation of the programme and the protocol for use.

The HTS is a computerized visual therapy system which is used by the subject in his or her home environment at a distance of 40 cm. The programme uses images that the subject has to fuse in order to perceive three dimensional (stereoscopic) images. These stereoscopic images are resolved using red/blue spectacles. The HTS was developed by Dr. Jeffrey Cooper and Rodney K. Bortel, and is used widely in the United States for patients with asthenopic symptoms [14,16,20].

A demonstration of the HTS was provided and subjects and parents were advised that they could contact the practitioner (WD) with any queries regarding the programme. Subjects were advised to perform 3-4 sessions per week, each session lasting approximately 15-20 minutes, similarly to that recommended by The Convergence Insufficiency Treatment Trial Group [16]. The parents were instructed to regularly supervise and ensure that their child was carrying out the procedure correctly. This was done using an instrument incorporated in the programme allowing the date, time and performance of the exercises to be reviewed. These reviews were also carried out on a weekly basis by the practitioner (WD).

A range of visual function tests were carried out pre and post treatment. The procedures for these tests have previously been described in detail [10].

All 134 subjects were reassessed after four weeks and the tests were repeated in the same order using exactly the same testing conditions as previously. Subjects with clinically significant refractive errors (≥ +1.00D hyperopia, ≤ -0.50D myopia, ≤ -1.00DC astigmatism or ≥ 1.00D anisometropa), wore his or her habitual spectacles during testing at both visits. Prismatic reading spectacles were not worn during testing at either visit.

### Reading speed and accuracy

Reading speed and accuracy were assessed using a standard Austrian test known as The Salzburg Reading Test. Age appropriate material suitable for each particular subject was selected and the test was conducted in a quiet room. The child was asked to start reading a prescribed section of text and the time taken to complete the task was measured with a stopwatch. In addition, the number of incorrect words read was noted and an error score calculated [21].

### Ocular Posture

A standard cover-uncover test and alternating cover test revealed the presence and direction of heterotropias and heterophorias at distance and near (5 m and 40 cm). The subject was asked to fixate an acuity appropriate Polatest target for three seconds before the eye was covered and uncovered. This was done for both eyes. A prism cover test was employed to assess the magnitude of the deviations present [22].

### Accommodation

Amplitude of accommodation was measured monocularly using the push-up method [23]. Binocular accommodative facility was assessed in cycles per minute using flipper lenses (+2.00/-2.00 D). This was repeated for one minute and the number of cycles noted [24].

Accommodative response to a target at a specific distance was assessed using Monocular Estimation Method (MEM) retinoscopy. The distance refractive error was fully corrected and a near target attached to the retinoscope at a distance of 40 cm. The subject was encouraged to read the text aloud while the retinoscopic reflex was observed. If the retinal reflex indicated a hyperopic or myopic state, plus or minus lenses, respectively, were added in 0.25 steps until neutrality was achieved [25].

### Convergence

Near point of convergence (NPC) was assessed using a standard protocol. Subjects were asked to fixate on the light of a pen torch while it was moved towards the subject's face and to report the point at which diplopia was first observed. The clinician also objectively assessed the point at which the subject lost fixation, when one eye deviated. The points at which the subject and the observer noticed a loss of fixation were noted [26,27].

Vergence facility was assessed in cycles per minute using a standard flip prism (3Δbase in/12Δbase out) that provides information about the condition and the speed of the vergence system [28]. Subjects were asked to fixate a small target on the Gulden stick at 40 cm and asked to try to keep the target single and clear. Prism (3Δ base-in) was introduced first and the subject was asked to report when it became single. When the target was single and clear the 12Δ base-out) was introduced. When the subject reported that the target was clear the prism was switched back to the 3Δ base in. This was repeated for one minute and the number of cycles was noted [28].

#### The accommodative convergence system of the eyes

The AC/A ratio was assessed by measuring the near phoria at 40 cm using the alternating cover test and prism bar. This was then repeated using -2.00D lenses in front of the eyes while the subject maintained fixation on the target at 40 cm. The AC/A ratio was calculated as the difference between the measured phoria with and without the -2.00D lenses, divided by two [29].

### Statistical analysis

All data were analysed for significance using SPSS 17.0 for Windows. All data were assessed for normality using the one sample Kolmogorov-Smirnov Test. Factorial analysis was used to evaluate between subject effects and within subject effects.

## Results

Treatment category was not found to be associated with age, spherical or cylindrical refractive error, reading time, reading error score or near point of convergence (p > 0.05 using one way ANOVA). The Kolmogorov-Smirnov Test for normality demonstrated a normal distribution in all groups for all measurements.

### Compliance

Compliance was assessed verbally, addressing both the parents and children. Both parents and subjects reported that spectacles were worn > 80% of time when undertaking near vision tasks. In addition, subjects prescribed the HTS were compliant at least 80% of the time.

### Before intervention

For all visual function measures there were no significant differences between the groups before intervention (one way analysis of variance [ANOVA] p > 0.05). Tables 2, 3, 4, 5 and 6 provide mean values for each visual function parameter before intervention.

**Table 2:** Mean total reading time for each subject group at the first and second visit

Group	Mean total reading time (seconds) ± SD		
	**First visit**	**Second visit**	**Mean difference (seconds) ± SD**

**Control group**	130.88 ± 61.46 (n = 32)	127.03 ± 60.59 (n = 32)	3.84 ± 4.04 (n = 32)

**HTS**	113.98 ± 48.83 (n = 51)	101.61 ± 37.53 (n = 51)	12.37 ± 16.22 (n = 51)

**Reading spectacles with 8Δ base-in**	108.49 ± 48.68 (n = 51)	87.00 ± 39.60 (n = 51)	21.49 ± 13.53 (n = 51)

**Table 3:** Mean reading error score for each subject group at the first and second visit

Group	Mean reading error score ± SD		
	**First visit**	**Second visit**	**Mean difference ± SD**

**Control group**	5.34 ± 3.5 (n = 32)	4.66 ± 2.9 (n = 32)	0.69 ± 1.20 (n = 32)

**HTS**	4.53 ± 3.06 (n = 51)	2.86 ± 1.9 (n = 51)	1.67 ± 1.90 (n = 51)

**Reading spectacles with 8Δ base-in**	4.92 ± 4.06 (n = 51)	2.12 ± 1.9 (n = 51)	2.80 ± 2.82 (n = 51)

**Table 4:** Mean amplitude of accommodation at each visit for each subject group

Group	Amplitude of accommodation (D) ± SD		
	**First visit**	**Second visit**	**Mean difference (D) ± SD**

**Control group**	12.66 ± 2.3 (n = 32)	12.97 ± 1.6 (n = 32)	0.31 ± 1.28 (n = 32)

**HTS**	11.86 ± 2.6 (n = 51)	12.88 ± 1.7 (n = 49)	1.02 ± 1.50 (n = 51)

**Reading spectacles with 8Δ base-in**	11.51 ± 2.5 (n = 51)	12.92 ± 1.5 (n = 51)	1.41 ± 1.37 (n = 51)

**Table 5:** Mean binocular accommodative facility test score at each visit for each subject group

Group	Binocular accommodative facility test score (cycles per minute) ± SD		
	**First visit**	**Second visit**	**Mean difference (cycles per minute) ± SD**

**Control group**	5.59 ± 3.2 (n = 30)	6.38 ± 2.5 (n = 30)	0.78 ± 1.58 (n = 32)

**HTS**	6.20 ± 3.9 (n = 51)	9.78 ± 3.4 (n = 51)	3.59 ± 2.98 (n = 51)

**Reading spectacles with 8 Δ base-in**	5.53 ± 2.9 (n = 51)	8.88 ± 2.9 (n = 51)	3.35 ± 2.11 (n = 51)

**Table 6:** Mean vergence facility test score at each visit for each subject group

Group	Vergence facility test score (cycles per minute) ± SD		
	**First visit**	**Second visit**	**Mean difference ± SD**

**Control group**	5.44 ± 3.7 (n = 30)	6.63 ± 3.7 (n = 30)	1.19 ± 1.63 (n = 32)

**HTS**	5.80 ± 4.6 (n = 51)	9.78 ± 3.8 (n = 51)	3.98 ± 3.83 (n = 51)

**Reading spectacles with 8 Δ** **base in**	4.96 ± 4.3 (n = 51)	8.96 ± 3.7 (n = 51)	4.00 ± 2.61 (n = 51)

Before intervention the mean total reading time was 130.88 ± 61.46 seconds in the control group, 113.98 ± 48.83 seconds in the HTS group and 108.49 ± 48.68 seconds in the prism spectacle group. The mean reading error score was 5.34 ± 3.5 in the control group, 4.53 ± 3.06 in the HTS group and 4.92 ± 4.06 in the prism spectacles group. The mean amplitude of accommodation was 12.66 ± 2.3D in the control group, 11.86 ± 2.6D in the HTS group and 11.51 ± 2.5 in the prism spectacle group. The mean binocular accommodative facility score was 5.59 ± 3.2 cycles per minute in the control group, 6.20 ± 3.9 cycles per minute in the HTS group and 5.53 ± 2.9 cycles per minute in the prism spectacle group. The mean vergence facility test score was 5.44 ± 3.7 cycles per minute in the control group, 5.80 ± 4.6 cycles per minute in the HTS group and 4.96 ± 4.3 cycles per minute in the prism spectacle group.

### Refractive error

Refractive error profiles (sphere, cylinder and spherical equivalent) were normally distributed (one-sample Kolmogorov-Smirnov Test for normality). Spherical equivalent refractive errors (sphere + cylinder/2) ranged from -2.13D to +4.63D.

### Factorial analysis

Factorial analyses demonstrated statistically significant changes between results obtained for visits 1 and 2 for near phoria, near point of convergence and MEM retinoscopy (within subjects effects [time]) (Table 7).

**Table 7:** Factorial analysis results

Outcome measure	Time × treatment (interaction effect)		Time (within subjects effects)		Treatment (between subjects effects)	
	**F (degrees of freedom)**	**p**	**F (degrees of freedom)**	**p**	**F (degrees of freedom)**	**p**

**Total reading time**	18.04 (2,131)	< 0.001	115.75 (1, 131)	< 0.001	4.17 (2,131)	0.018

**Reading error score**	9.58 (2,131)	< 0.001	79.23 (1, 131)	< 0.001	3.14 (2,131)	0.047

**Amplitude of accommodation**	6.04 (2,131)	0.003	54.21 (1,131)	< 0.001	0.88 (2,131)	0.417

**Binocular accommodative facility**	15.56 (2,131)	< 0.001	148.32 (1, 131)	< 0.001	4.46 (2,131)	0.013

**Vergence facility**	10.81 (2,131)	< 0.001	134.72 (1,131)	< 0.001	2.19 (2,131)	0.116

**Near phoria**	0.40 (2,131)	0.673	31.71 (1,131)	< 0.001	0.783 (2,131)	0.459

**Near point of convergence**	2.41 (1,131)	0.094	18.01 (1,131)	< 0.001	0.606 (2,131)	0.547

**MEM retinoscopy**	0.223 (1,126)	0.800	6.59 (1,126)	0.011	0.768 (2,126)	0.466

Significant interaction effects were noted for the following outcome measures; total reading time, reading error score, amplitude of accommodation, binocular accommodative facility and vergence facility (Table 7). Further examination of simple main effects of the within factor (time) at each level of the between factors (treatment) indicated that significant improvements were noted between visits 1 and 2 for total reading time, reading error score, amplitude of accommodation, binocular accommodative facility and vergence facility for the prism spectacles and HTS groups (p < 0.05). The only significant effect noted between visits 1 and 2 for the control group was vergence facility (p = 0.026).

**Figure 2:**
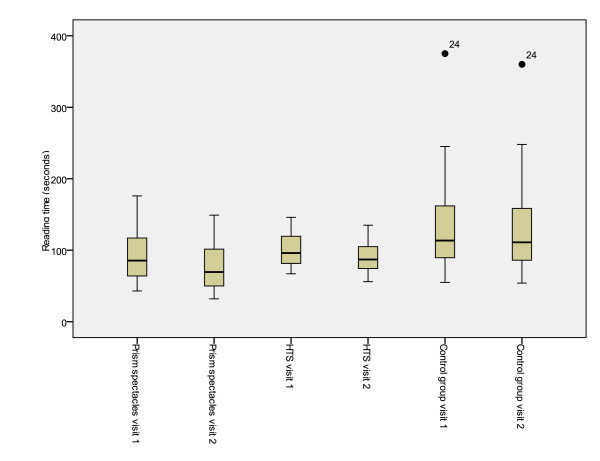
**Box plots of total reading time at first and second visits for each subject group**. The top of the box represents the 75^th ^percentile, the bottom of the box represents the 25^th ^percentile and the middle line represents the 50^th ^percentile. The whiskers represent the highest and lowest values (excluding outliers). Outliers are represented by the closed circles. Asterisks represent extreme values.

**Figure 3:**
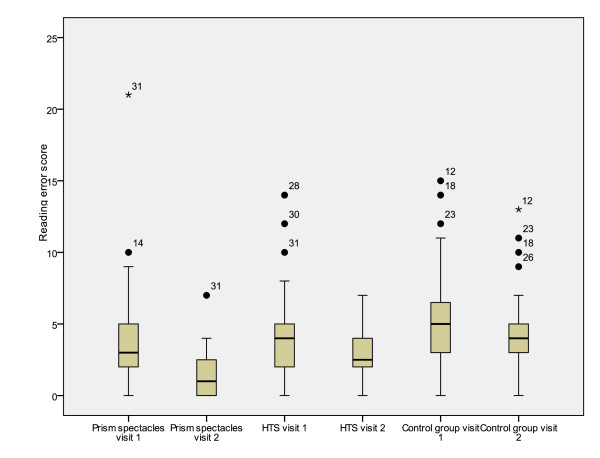
**Box plots of reading error scores at first and second visits for each subject group**. The top of the box represents the 75^th ^percentile, the bottom of the box represents the 25^th ^percentile and the middle line represents the 50^th ^percentile. The whiskers represent the highest and lowest values (excluding outliers). Outliers are represented by the closed circles. Asterisks represent extreme values.

**Figure 4:**
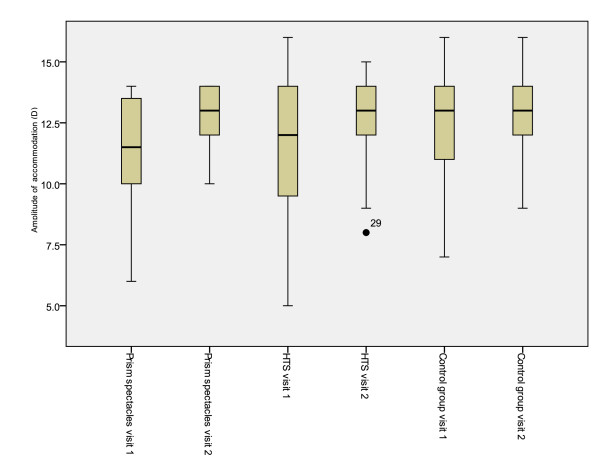
**Box plots of amplitude of accommodation at first and second visits for each subject group**. The top of the box represents the 75^th ^percentile, the bottom of the box represents the 25^th ^percentile and the middle line represents the 50^th ^percentile. The whiskers represent the highest and lowest values (excluding outliers). Outliers are represented by the closed circles. Asterisks represent extreme values.

**Figure 5:**
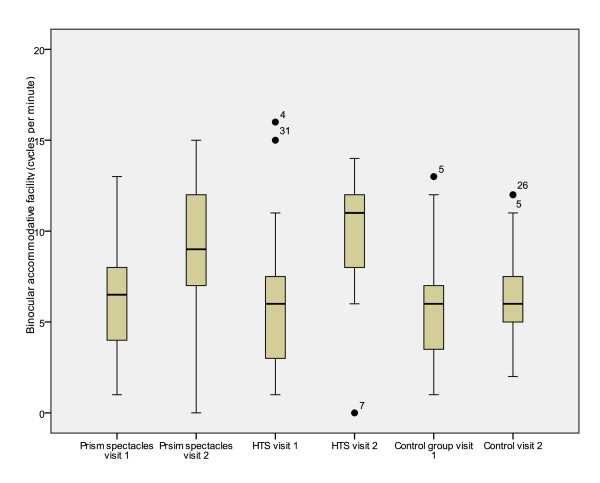
**Box plots of binocular accommodative facility at first and second visits for each subject group**. The top of the box represents the 75^th ^percentile, the bottom of the box represents the 25^th ^percentile and the middle line represents the 50^th ^percentile. The whiskers represent the highest and lowest values (excluding outliers). Outliers are represented by the closed circles. Asterisks represent extreme values.

**Figure 6:**
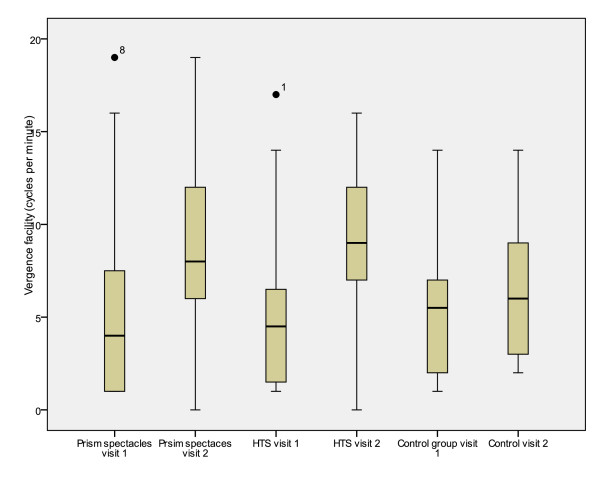
**Box plots of vergence facility at first and second visits for each subject group**. The top of the box represents the 75^th ^percentile, the bottom of the box represents the 25^th ^percentile and the middle line represents the 50^th ^percentile. The whiskers represent the highest and lowest values (excluding outliers). Outliers are represented by the closed circles. Asterisks represent extreme values.

Significant differences were also demonstrated between treatment groups for total reading time, reading error score and binocular accommodative facility (between subjects effects [treatment]) (Table 7). Mean values for the first and second visits and mean differences between visits are presented in tables 2, 3, 4, 5 and 6. Figures 2, 3, 4, 5 and 6 represent the changes in outcome measures with each treatment.

Raw data are available as an additional file (Additional file 1).

## Discussion

Reading and writing are the most important learning performance indicators in the early school years. Whilst it is now recognised that an apparently underachieving child may not lack intellectual prowess or ability but that there may be functional or psychological issues that impede learning, there is still a paucity of basic investigations to determine how some of these problems can be addressed or alleviated. A diagnosis of a specific reading difficulty is a significant concern for parents and children and it is possible that basic binocular visual problems are overlooked amidst the investigations. Even when visual problems are diagnosed, if these are vergence-based, prismatic correction is not routinely prescribed. Too few studies have compared the effect of a relatively high prismatic correction with other treatment modalities on children with visual problems such as CI which affects reading performance.

All measurements in this study were obtained using the same techniques under uniform, controlled conditions and conducted by the same practitioner ensuring the avoidance of practitioner variability.

Improvements in five outcome measures (reading speed, reading error score, amplitude of accommodation, vergence facility, and binocular accommodative facility) were noted between results obtained for visits 1 and 2. In addition, significant differences between treatment groups were observed for reading speed and reading error score, with subjects with the prism spectacles showing the greatest improvements. Whilst statistical significance may not always manifest as significant in the clinical realm as the changes may be too subtle, this study indicates which visual functions were affected by the various treatment options and, most importantly from the perspective of the scholastic attainment, which showed improvements in reading. Reading speed is an important outcome measure as it reflects clinically significant changes in results in addition to statistically significant results. Changes in reading speed provide data that is meaningful and highly applicable to daily living tasks.

Scheiman et al. [18] suggest that base-in reading spectacles are not an appropriate treatment option for CI as they were no more effective than placebo spectacles at improving near point of convergence, fusional vergence or reducing asthenopic symptoms. However, the present study found that prism spectacles with 8Δ base-in significantly improved both reading speed and reading errors scores. Most importantly, this was the only treatment that improved both total reading time and error score which is most pertinent to scholastic achievement. The discrepancy in findings between this study and previous work [18] may be explained by the differences in the study populations, differences in the prescribed spectacles or type of outcome measures used. In the present study subjects were between 7 and 14 years of age. The previous study was conducted on a group ranging in age from 9 to 18 years. It may be possible that the slightly younger cohort in this study was more amenable to treatment as they had not yet reached the end of the sensitive period for visual development. Differences may also be attributed to the size of the prism used for treatment. Scheiman et al [18] based the size of the prism prescribed on Sheard's criterion and this resulted in a mean value of 4.14Δ. It is possible that the larger amount of prism prescribed in the present study allowed comfortable clear single vision to be obtained for longer periods. It is interesting to note that these improvements in reading speed and reduction in reading error scores were obtained in the absence of the base-in spectacles. This may be due to the spectacles stimulating an improvement in fusional reserves demonstrated by the improvements in vergence facility tests. It is unlikely that that the improvements noted in reading ability were due to a learning effect as these tests were performed on only two occasions and the tests included both real and pseudo words.

It may be argued that the length of time between visits was relatively short in comparison to other studies [18]. The four week period was chosen to ensure that the subjects were monitored carefully, that the treatment did not have a detrimental effect on visual function and that subjects did not tire of the treatment and cease its application. In addition, the authors were aware that the reading ability and speed of school children may increase significantly over a longer time period purely due to educational development over time. Due to the short time period used, any improvements in visual function are more likely to be attributable to the CI treatment rather than to a general improvement in reading skills. The four week period between visits allowed sufficient time for adaptation to the new spectacles.

Questionnaires developed for use in children with CI have been reported [30,31]. Scheiman et al. [18] demonstrated no improvement in the Convergence Insufficiency Symptom Survey score with the use of base-in prism reading glasses in a group of children aged 9-18 years. The use of questionnaires in the present study would have been of limited value due to the younger cohort in the present study compared to that of Scheiman et al. [18] and the subjective nature of the outcome measure: symptoms and their reporting can be vague and unreliable particularly with younger subjects.

In accordance with previous studies the HTS produced an improvement in various measures of visual function (including reading error score, amplitude of accommodation, binocular accommodative facility and vergence facility) [14,16,20]. Although patient compliance may pose a concern with this form of treatment, this was monitored by parents and the practitioner throughout the present study using data generated by the HTS. Results of the present study suggest that the use of the HTS provides a useful alternative to base-in prism spectacles where the optical correction is not acceptable to patients. Some computer based vision therapy systems may prove difficult and confusing for children, especially those of younger age, and the relatively time consuming nature of the treatment may cause children and parents to give up easily and not to persist with treatment if improvements in visual function are not immediately noticeable. It is postulated that compliance with spectacle treatment of CI with base-in prism reading spectacles was good as the treatment provides immediate relief for children struggling with near vision tasks. However, it was only possible to assess this based on parent's and subject's opinions.

All measurements were obtained by the same examiner (WD) to avoid practitioner variations. The examiner did not enquire about what treatment had been given, when the subject returned for the second visit. Whilst it cannot be ruled out that the examiner may have been aware in some cases to which treatment group individual subjects belonged, the major outcome variables: reading speed and accuracy, were measured with a test that is as objective as possible, minimising the influence of any bias. The latter point notwithstanding, where the experiment is not conducted as a randomised clinical trial, bias cannot be ruled out. Potential sources of bias in this investigation are most likely to be related to the reasons why a given treatment option was chosen. Such preferences could be affected by certain functional factors (e.g. refractive status, vergence capacity), visual demands (e.g. time spent doing near work) or they may stem from inherent psychological characteristics that may influence the choice of a particular treatment option (e.g. a preference for or aversion to the wearing of spectacles). Whilst, it is not possible to investigate all these potential sources of bias in the present study, functional factors that could be determined clinically were measured and it was assumed that the demands on the visual system did not vary significantly from one treatment group to another. However, subtle functional differences that may not be manifest clinically and that may influence choice cannot be dismissed and may form the basis of further investigations in this area. Likewise, questionnaire based studies that probe time spent on near activities and on the types of activities may reveal further sources of bias. It is difficult to speculate on potential psychological reasons for choice of treatment as these are beyond the scope of this study, whilst the subjects were assessed by an educational psychologist and no data relating to psychological testing was accessible. This clinical study provides useful and seminal preliminary data that warrant further investigation, that ideally should be followed up in a randomised controlled trial.

Treatment of CI in children with reading difficulties is important to prevent these visual anomalies from further compounding educational issues associated with the reading difficulties. Reading difficulties associated with binocular vision anomalies are highly likely to lead to reduced educational attainment [1]. This may prevent a child from progressing through school with their peer group and would cause unnecessary social exclusion.

## Conclusions

The importance of diagnosing and treating a problem like CI that has the potential to lead to significant problems that could severely undermine scholastic achievement should not be underestimated. Further investigations into the contribution of CI in reading, writing and learning difficulties are needed to define the extent of the problem and to consolidate and harmonise treatment practices. The present study demonstrates that prismatic correction offers an effective treatment option for children with CI and reading difficulties that arise from causes not linked to intellectual ability.

## Competing interests

The authors declare that they have no competing interests.

## Authors' contributions

All authors read and approved the final version of the manuscript. All authors participated in the design of the study, data analysis and preparation of the manuscript. All data were collected by WD.

## Pre-publication history

The pre-publication history for this paper can be accessed here:

http://www.biomedcentral.com/1471-2415/11/21/prepub

## Supplementary Material

Additional file 1 **Raw data**. Additional file 1 contains all the raw data from the study. Click here for file
